# Mechanism of Isotropic Behavior in Titanium Alloy Plates Formed by Axial Closed Die Rolling

**DOI:** 10.3390/ma18112528

**Published:** 2025-05-27

**Authors:** Jungang Nan, Dong Liu, Yonghao Zhang, Yu Zhang, Jianguo Wang

**Affiliations:** 1 School of Materials Science and Engineering, Northwestern Polytechnical University, Xi’an 710072, China; nanjgang6011@mail.nwpu.edu.cn (J.N.); liudong@nwpu.edu.cn (D.L.); 2023261259@nwpu.edu.cn (Y.Z.); zy0429@mail.nwpu.edu.cn (Y.Z.); 2 Anhui Hangon Ultrafine Metal Technology Co., Ltd., Suzhou 234200, China

**Keywords:** titanium alloy, ACDR, EBSD, deformation texture, isotropic

## Abstract

The torsional behavior during the deformation process of the axial closed die rolling the axial closed rolling (ACDR) forming is studied in this paper using a numerical simulation technique on TC11 titanium alloy. The axial and radial pinch angles, as well as the degree of specimen torsion, increased with the amount of deformation. The orientation distribution function (ODF) maps of the α-phase and β-phase were obtained by Electron Back Scatter Diffraction (EBSD) treatment of the TC11 titanium alloy. It can be noticed that there were different types of texture with different strengths in the ACDR samples, and in the *xz* and *yz* planes, textures in the direction of the column were predominantly of {0001} <2$\bar{1}\bar{1}$0> and {01$\bar{1}$0} <2$\bar{1}\bar{1}$0>; the weaker the texture was, the closer to the edge of the sample. In the *xy* plane, the texture structure was mainly distributed along the cone direction, and the textures were {$\bar{1}$2$\bar{1}$0} <10$\bar{1}$0> and {01$\bar{1}$0} <2$\bar{1}\bar{1}$0>. However, the closer to the edge position of the specimen, the higher the intensity of the texture, and the texture was {1$\bar{2}$1$\bar{2}$} <1$\bar{2}$16>. The β-phase is mainly distributed as {001} <100>, {110} <1$\bar{1}$0>, and {110} <001> textures within the specimen, and the texture strength is about 8.5 times. However, owing to the small proportion of the β-phase content in the specimen, the distribution pattern of its texture has a weak impact on the texture distribution of the overall specimen. A high degree of isotropy in the radial and tangential tensile properties, with a strength isotropy of over 99 percent and a plasticity isotropy of over 95 percent, resulted from the distribution of texture types with varying strengths and orientations within the ACDR specimens, which weakened the TC11 discs’ overall orientation.

## 1. Introduction

Due to the exceptional creep resistance below 500 °C and high room-temperature strength, TC11 titanium alloy finds extensive application in aero-engine components like compressor disks, drums, and blades [1]. The morphological properties of its initiating phase and the distribution of the deformation wave have a synergistic effect on its mechanical properties. It has been indicated that although the lamellar phase structure of the laminate has high strength and resistance to cracking, its parallel lath structure tends to result in significant anisotropy and a significant loss of plasticity along the longitudinal direction of the lath. On the other hand, the equiaxial α-phase structure has better plastic reserves and superior durability properties while maintaining strength because of its uniform grain orientation distribution. Consequently, the textures generated during the forging process aggravated the anisotropy of the material [2,3].

Anisotropy in mechanical properties results from the unequal ease of initiation of slip systems with different orientations when the incipient α-phase is preferentially oriented in a particular direction after deformation. This is especially important in titanium alloy disks with strong texture. Gey et al. analyzed the texture evolution of α- and β-phases in hot-rolled Ti-64 alloy by EBSD and discovered that α-phase basal slip dominated the formation of rolled texture and β-phase dynamic recrystallization contributed to the randomization of the orientation, resulting in macro-mechanical anisotropy [4]. Dynamic recrystallization of the pristine β-phase interacts with static restitution of the α-phase, according to Li’s investigation of the static annealing process of the TC11 alloy in the hot-worked state. The gradient of the orientation difference and the difference in grain boundary mobility are the primary causes of texture anisotropy [5]. Dixit et al. examined how the temperature and rate of cooling of solid solutions affected the texture of laminated, isometric, and bimodal Ti-6Al-4V alloys. The incipient isometric α exhibits a rolling texture that vanishes with the recrystallization process. A <10$\bar{1}$0> texture with moderate strength, the α slats undergo transformation to create a strong columnar surface texture, and the high temperature of the solid solution encourages the β-phase to change into the α-phase [6,7]. Lu used hot compression experiments to examine the texture evolution in the Ti-6Al-3Nb-2Zr-1Mo alloy’s α + β/β-phase region. Discontinuous Dynamic Recrystallization (CDRX) and Continuous Dynamic Recrystallization (DDRX) coexist at the deformation temperature of 900 °C, with DDRX predominating and decreasing the texture density; at temperatures between 980 °C and 1020 °C, CDRX is weakened, and parallel precipitation of α strengthens the laths. <0001>//Normal direction (ND) basal texture, a significant contributing factor to the texture variation, is the gradient change of the deformation storage energy [8]. In the forging process of the Ti-5553 alloy, Wang discovered that the growth anisotropy of the α-phase results in varying texture strengths. The weak texture region displays a staggered mesh structure, while the strong texture region’s α lamellae tend to be arranged in parallel with the lamellae spacing reduced by 30 to 50 percent [9].

**Figure 1:**
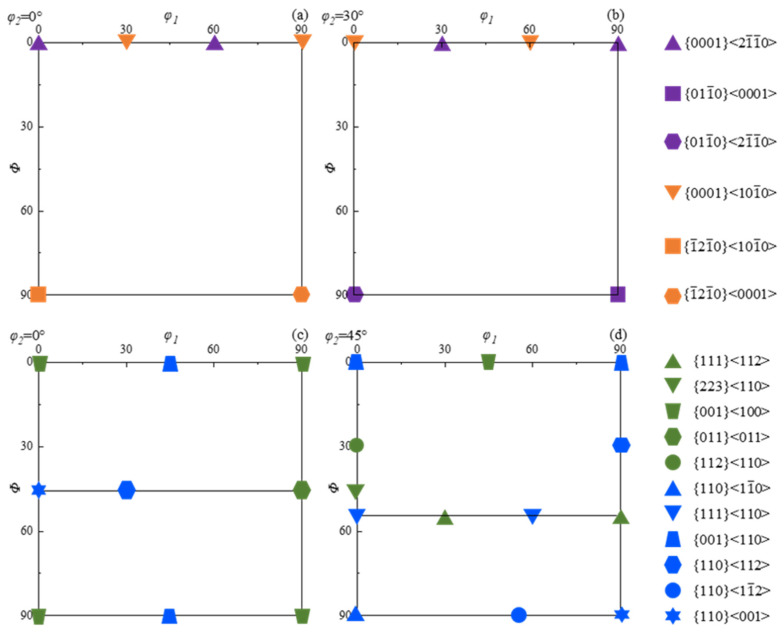
Deformation textures of titanium alloys [12,13]: (**a**) *φ*_2_ = 0°, α-phase; (**b**) *φ*_2_ = 30°, α-phase; (**c**) *φ*_2_ = 0°, β-phase; and (**d**) *φ*_2_ = 45°, β-phase.

Through repeated forging and forming, texture effects on property anisotropy in titanium alloys are typically weakened. Li compared the effect of one-way forging and two-way drawing on the texture of Ti-6Al-4V, which is predominantly a basal slip forming a {0001} basal texture in the low-strain area, {11$\bar{2}$0} <10$\bar{1}$0> in the high-strain area, and {11$\bar{2}$2} <10$\bar{1}$1> in the stress concentration region. The orientation distribution is impacted by dislocation density gradients and slip coefficient activation differences, while multidirectional forging homogenizes the deformation texture. Additionally, α/β-phase-coordinated deformation reduces the texture strength [10]. Kang investigated the use of a T-shaped multi-pass equal-channel angular pressing (ECAP) coordinated cold rolling process on industrially pure titanium. This process creates high-density dislocations and subgrain boundaries by causing intense plastic deformation through a continuous shear path. ECAP’s cumulative strain strengthens the {10$\bar{1}$0}//Roll direction (RD) shear texture, while cold rolling exacerbates the grains’ elongation orientation [11]. The ODF maps of the sample surface can be obtained using the EBSD technique by rotating the sample successively by angles *φ*_1_, *Φ*, and *φ*_2_ to make the crystal coordinate system consistent with the sample coordinate system. The Euler angles (*φ*_1_–*Φ*–*φ*_2_), as defined by Bunge, can be used to characterize the texture [12]. In the ODF diagrams of titanium alloys, the most common texture types of the α-phase are mainly distributed at *φ*_2_ = 0° and *φ*_2_ = 30°, and the most common texture types of the β-phase are distributed at *φ*_2_ = 0° and *φ*_2_ = 45°. The typical textures of titanium alloys that are currently created during deformation are displayed in Figure 1 [12,13].

Duplex titanium alloy is a typical “process-tissue-property” sensitive material, meaning that its mechanical properties and microstructure characteristics are intricately coupled. The process parameters during the thermal deformation process control the dynamic recrystallization, phase transformation process, and other organizational evolution mechanisms, which collectively make up the main factors influencing the material’s mechanical properties. They also determine the morphology, size, and content distribution of the α/β-phases and help form a particular crystallographic texture during the deformation process. Anisotropy in titanium alloys can be successfully decreased and specimen uniformity enhanced by using the composite deformation mode, optimizing the deformation parameters and designing a gradient microstructure.

By effectively combining compression and torsion deformation, the axial closed rolling forming [14,15,16] technology that will be employed in this paper creates a new kind of industrial-grade forming technology. In the ACDR molding process, the upper die assembly is actively pressed down and the lower die assembly actively rotates the blank. At the same time, the upper die assembly contacts the blank and rotates passively under the action of friction, resulting in continuous local molding of the upper workpiece surface [14]. The mechanism underlying the distribution of microstructure orientation and the anisotropy of titanium alloy properties under this deformation technology has not yet been documented. Using TC11 titanium alloy as the research object, the ACDR technology is used to investigate the texture type and distribution strength of the titanium alloy under compression and torsion deformation conditions that affect anisotropy performance. This is especially crucial for accurate material flow line control and forging quality.

## 2. Material and Methods

### 2.1. Test Materials

**Figure 2:**
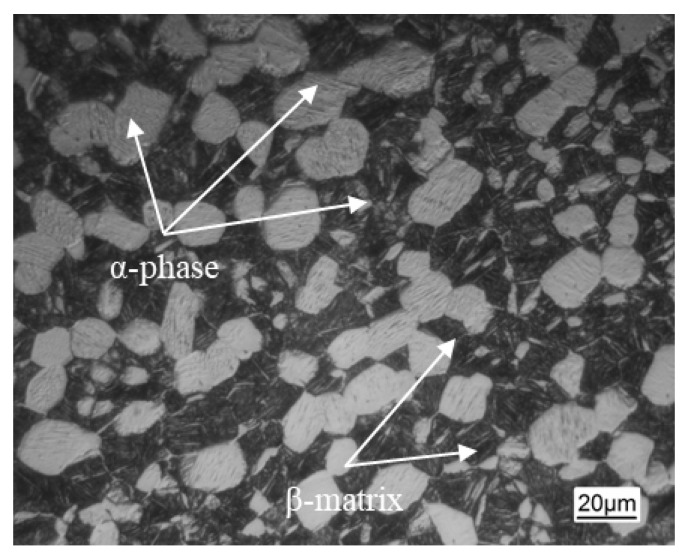
Initial microstructure of the TC11 titanium alloy.

**Figure 3:**
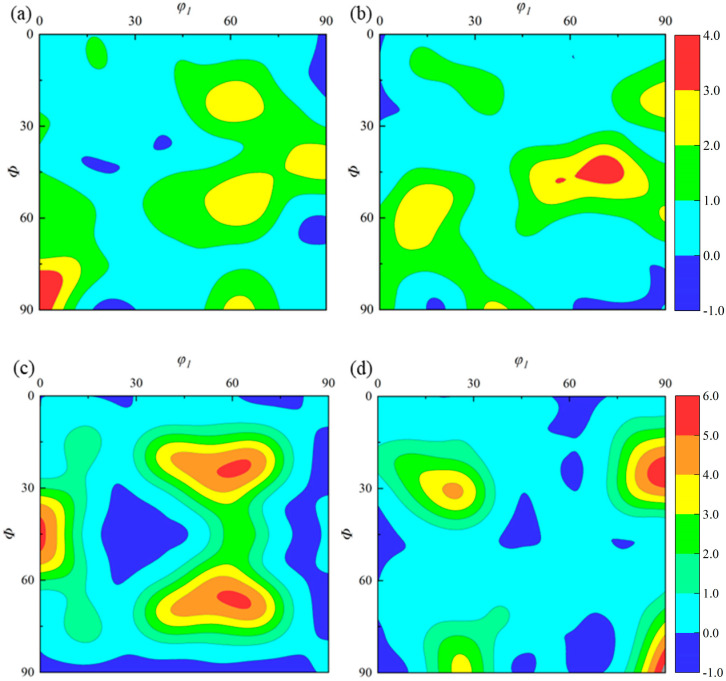
Initial ODF distribution of the TC11 titanium alloy: (**a**) *φ*_2_ = 0°, α-phase; (**b**) *φ*_2_ = 30°, α-phase; (**c**) *φ*_2_ = 0°, β-phase; and (**d**) *φ*_2_ = 45°, β-phase.

In this thesis, TC11 titanium alloy hot-rolled bars with a diameter of 300 mm were used from Baoji Xigong Titanium Alloy Products Co., Ltd. (Baoji, China) and their chemical compositions are listed in Table 1. Metallurgical observation was used to determine the TC11 titanium alloy’s β-transition temperature, which came out to be 1010 ± 5 °C. The initial state’s microstructure of the longitudinal region is displayed in Figure 2. Most of the α-phase exhibits a short rod-like morphology and is homogeneously distributed on the β-matrix. The initial α-phase has an average size of 19 ± 0.5 μm and a content of roughly 27 percent, as determined by Image Pro-Plus software 6.0. It can be observed from the ODF plots of the titanium alloy’s original organization in Figure 3 that the specimen’s texture strength and content are both low. The α-phase textures are mainly distributed in {$\bar{1}$2$\bar{1}$0} <10$\bar{1}$0> and {1$\bar{1}$0$\bar{2}$} <01$\bar{11}$>, with the maximum texture strength of about 4.04, and the β-phase textures are mainly concentrated in {110} <001> and {110} <112>, whose maximum textures strength is about 6.01.

### 2.2. Test Scheme

**Figure 4:**
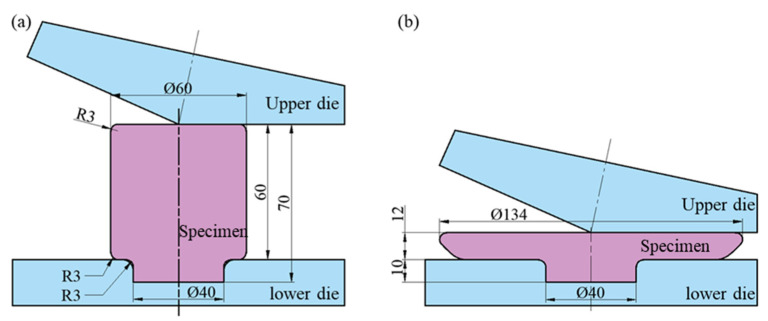
Schematic diagrams of the titanium alloy specimens before (**a**) and after (**b**) ACDR deformation.

The TC11 titanium alloy’s ACDR deformation test programs [14] are displayed in Table 2. The double annealing heat treatment was followed by the mechanical property test and microstructure observation. The specimen was heated to 950 °C, held for 1.5 h, and then allowed to cool naturally. Thereafter, it was heated to 550 °C, held for 4 h, and allowed to cool naturally to room temperature. Figure 4 displays the schematic diagrams of the specimens in ACDR both before and after deformation.

### 2.3. Microstructure and Performance Test Sampling Schemes

**Figure 5:**
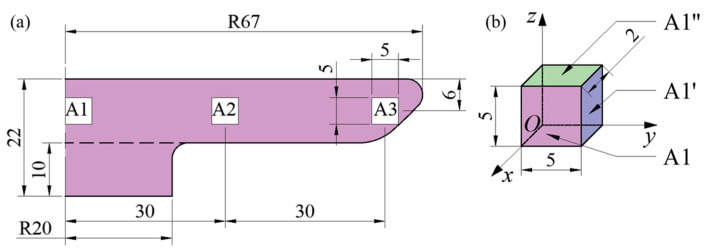
(**a**) Typical position of specimen H/2 height and (**b**) detailed characterization of A1 specimen.

After the heat-treated TC11 specimens were cut longitudinally, three sets of 5 × 5 × 2 mm specimens were taken out at the specimens’ H/2 height position and labeled A1, A2, and A3 from the center of the specimen to the edge. It was determined that the A1, A2, and A3 planes were the specimens’ *yz* planes, the A1’, A2’, and A3’ planes were their *xz* planes, and the A1”, A1”, A2”, and A3” surfaces were their *xy* surfaces. The specimens’ *xy*, *xz*, and *yz* planes were analyzed by EBSD. Figure 5 displays the sampling site and three-dimensional schematic diagram.

The 5 × 5 mm sections were polished with 80#, 200#, 800#, and 2000# sandpaper, followed by polishing solution (SiO_2_ aqueous solution/H_2_O_2_ = 90/10) to gain the mirror effect. This was followed by electrolytic polishing in an electrolytic solution of liquid nitrogen (HClO_4_/C_4_HO/CH_3_OH = 6/30/64). Using a titanium plate as the cathode and a polishing voltage of 20V, the electrolytic polishing process took 40–60 s to complete. An AMBER-type focused-ion double-beam electron microscope with an EBSD probe, a suitable scanning area, and a scanning step size of 0.02 μm was used to conduct the EBSD test. Following testing, the specimens’ crystallographic data were gathered and examined using HKL’s professional EBSD analysis software, Channel 5 (2019 v5.12).

**Figure 6:**
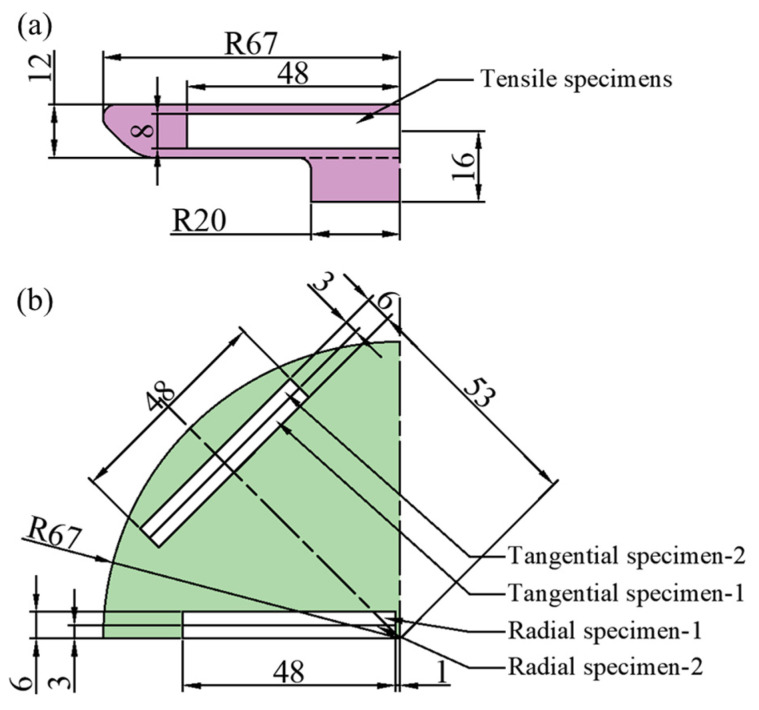
(**a**) Main view and (**b**) top view of the performance sampling scheme.

**Figure 7:**
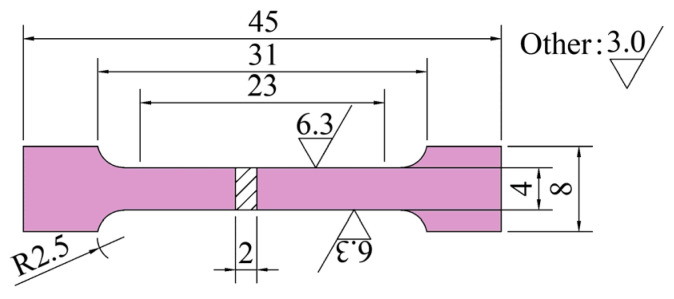
Size of standard lamellar tensile specimen.

The mechanical properties of the deformed specimens are tested in order to determine the law of the organization’s influence on the mechanical properties. The tensile property specimen sampling plan is displayed in Figure 6, employing 48 × 8 × 3 mm blank specimens that were machined into 45 × 8 × 2 mm sheet tensile specimens in compliance with the GB/T 228-2021 standard [17], as illustrated in Figure 7. Using an INSTRON 3382 universal testing machine (Norwood, MA, USA), the room temperature (25 °C) tensile test was conducted at a tensile strain rate of 0.0005 s^−1^.

## 3. Experimental Results and Discussion

### 3.1. Reversal of Behavioral Characterization

**Figure 8:**
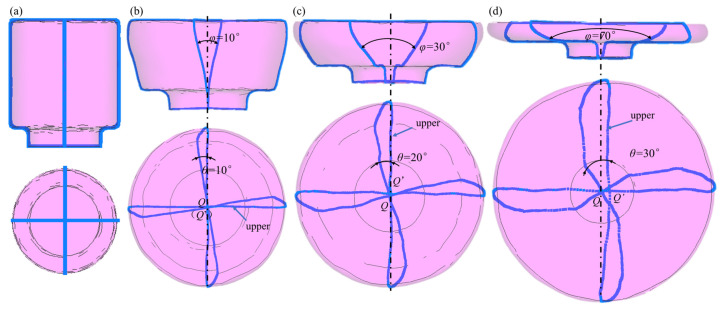
Torsional streamlines distribution on the surface of the ACDR specimen at (**a**) undeformed; (**b**) *η* = 40%; (**c**) *η* = 60%; and (**d**) *η* = 80%.

When torsion is added, the titanium alloy microstructure’s morphology and dimensions differ greatly from those of upsetting and forming. Two mutually perpendicular tracking lines are embedded on the specimen’s surface during the numerical simulation process to examine the torsion behavior of the TC11 titanium alloy during the ACDR, as illustrated in Figure 8a, to examine how the under pressure affects the axial rolling and forming process’s torsion behavior. When titanium alloy discs are formed using ACDR, the specimen creates a local plastic deformation zone in the height direction due to the local contact between the upper conical angle die and the upper surface of the specimen. This is different from the traditional forging and forming of the flow line. The TC11 titanium alloy’s torsional flow lines at a deformation temperature of 980 °C and various under-pressures are displayed in Figure 8b–d, where *Q*’ and *Q* stand for the geometrical centers of the specimen’s upper and lower surfaces, respectively, φ for the degree of axial flow line torsion, and *θ* for the degree of radial flow line torsion.

The number of torsion turns and the specimen’s torsion angle prior to deformation are the primary determinants of the degree of plastic deformation, according to the uniaxial torsion molding process. Equation (1) [14], when combined with the ACDR forming principle, can be used to express the number of rotational turns of the lower mold and the specimen’s degree of torsion.

(1) $$\sum =\frac{\omega \eta H}{\nu }$$

where *H* (mm) is the specimen height, *v* (mm/s) is the upper mold under pressing rate, *ω* (r/s) is the lower mold rotation speed, *η* (%) is the axial depression ratio, and *∑* is the number of rotations. The corresponding rotational circles are 4, 6, and 8, respectively, when the specimen deformation is 40%, 60%, and 80%.

As manifested in Figure 8b, the plastic deformation zone passes through the specimen’s deformation height at *η* = 40% and the specimen’s axial pinch angle *φ* = 10°. On the specimen’s upper surface, the streamlines continue to exhibit a linear distribution, but the streamlines on the lower surface exhibit some bending. When the intersection of the upper and lower surface streamlines converges in the surface geometric center at the corresponding radial streamline angle *θ* = 10°, it means that the specimen’s plastic deformation has just been transferred to its lower surface and that its overall degree of torsion is low.

The axial streamline angle shifts to *φ* = 30°, the plastic deformation zone reaches the specimen positioning table, and the entire height layer of the specimen has undergone plastic deformation when the amount of deformation reaches *η* = 60 percent (Figure 8c). The radial streamlines on the specimen’s lower surface bend more greatly, while the streamlines on the upper surface undergo a slight amount of bending deformation. Additionally, there is a slight deviation between the intersection points of the streamlines on the upper and lower surfaces. The radial streamline’s angle is currently *θ* = 20°.

Maintaining the deformation increase to *η* = 80 percent, as illustrated in Figure 8d, when the specimen’s axial streamline pinch angle is raised to 70°, parabolic lines appear in the streamlines throughout the specimen’s whole deformation height zone. At this point, the angle of clamping of the radial streamlines is *θ* = 30°. At the same time, the streamlines on the specimen’s lower surface exhibit a greater degree of bending, and the streamlines on its upper surface also seem bent, with a large curvature. The streamlines’ intersection point is also far from the geometric center.

The axial closed rolling forming is an inhomogeneous deformation mode that falls under the category of volumetric incremental forming. When the axial compression amount is small, the number of specimen rotation circles is small, the degree of torsion is small, and the radial (*θ*) and axial pinch angle (*φ*) are 10°; at this time, the deformation zone has not yet penetrated through the whole specimen. With the increase of the deformation amount, the specimen rotation circle increases, the degree of radial streamline torsion increases steadily, *θ* increases to 20°, the degree of axial streamline torsion increases rapidly, φ increases to 30°, and the height of the undeformed zone gradually decreases. At the same time, as the deformation continues to increase, the number of specimen rotation circles increases, the degree of torsional deformation is increasing, the intersection of radial streamlines on the upper surface has deviated from the geometric center, θ increases to 30°, and the degree of bending of radial streamlines on the lower surface rises. The deformation zone completely covers the height of the specimen, and at this time, φ is about 70°. According to certain studies [18,19], the combined effect of compression and torsion deformation can improve the microstructure of the titanium alloy.

### 3.2. EBSD Characterization and Analysis of the Specimens

**Figure 9:**
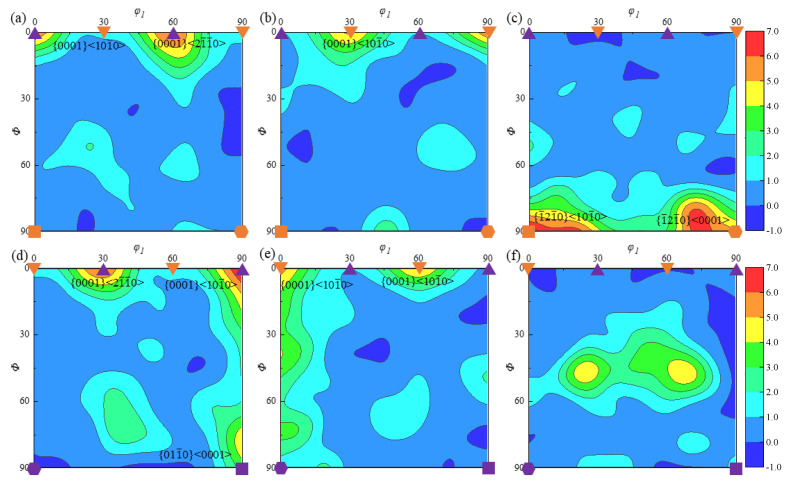
ODF plots of the primary α-phase at typical position A1 in (**a**) *φ*_2_ = 0°, *xz* plane; (**b**) *φ*_2_ = 0°, *yz* plane; (**c**) *φ*_2_ = 0°, *xy* plane; (**d**) *φ*_2_ = 30°, *xz* plane; (**e**) *φ*_2_ = 30°, *yz* plane; and (**f**) *φ*_2_ = 30°, *xy* plane.

Instantaneous positive strain and instantaneous shear strain at the specimen’s middle height position do not exhibit a cyclic variation rule during the ACDR molding process, as per the literature [20]. Instead, they both exhibit linear growth and higher values; the average grain size after deformation is about 11.7 μm. The texture of the three planes of α-phase and β-phase at the position of specimen A1 can therefore be more precisely analyzed using the Orientation Distribution Function (ODF) plot and the EBSD processing of the corresponding position. The red color indicates the maximum texture strength, as seen in Figure 9. As illustrated in Figure 9a, the maximum strength of the α-phase texture in the {0001} <2$\bar{1}\bar{1}$0> direction in the *xz* plane is 6.28 at *φ*_2_ = 0°. As seen in Figure 9b, the α-phase texture is more noticeable in the {0001} <10$\bar{1}$0> direction on the *yz* plane, reaching a maximum texture strength of 5.57. As seen in Figure 9c, the *xy* plane contains two stronger textures, {$\bar{1}$2$\bar{1}$0} <10$\bar{1}$0> and {$\bar{1}$2$\bar{1}$0} <0001>, with strengths of 8.28 and 6.97 times, respectively.

In the *xz* plane, when *φ*_2_ = 30°, as illustrated in Figure 9d, the α texture near {0001} <2$\bar{1}\bar{1}$0> has a maximum strength of 7.4. Texture {01$\bar{1}$0} <0001> strength is 5.16. According to Figure 9e, the strongest texture near {0001} <10$\bar{1}$0> is roughly 4.92 in the *yz* plane, and the strongest texture {0001} <2$\bar{1}\bar{1}$0> is 5.58. Other weaker textures have a texture strength of roughly 4.92 times and are evenly distributed on the Φ-axis. Other texture types with texture strengths of 5.53 and 5.03 times can be seen in the *xy* plane at (25–45–30°) and (70–45–30°), as illustrated in Figure 9f.

**Figure 10:**
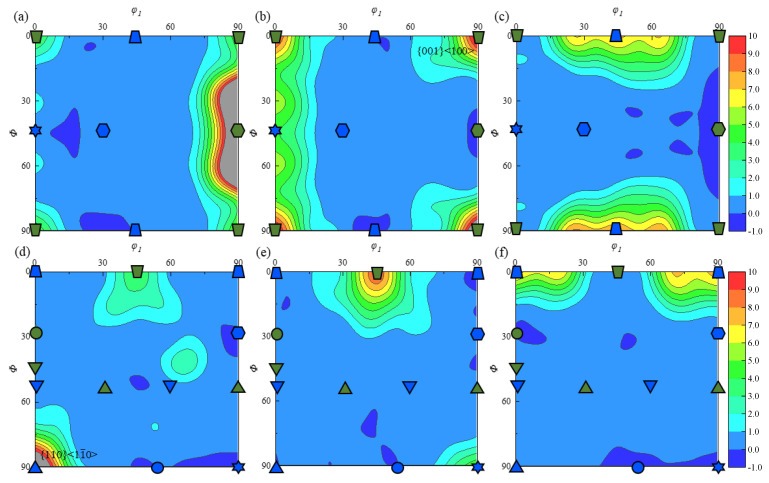
ODF plots of the β-phase at typical position A1 in (**a**) *φ*_2_ = 0°, *xz* plane; (**b**) *φ*_2_= 0°, *yz* plane; (**c**) *φ*_2_ = 0°, *xy* plane; (**d**) *φ*_2_ = 45°, *xz* plane; (**e**) *φ*_2_ = 45°, *yz* plane; and (**f**) *φ*_2_ = 45°, *xy* plane.

As for the β-phases, *φ*_2_ = 0° and *φ*_2_ = 45° in three planes at position A1 of the TC11 titanium alloy disk member; Figure 10 displays the most pertinent orientation distribution functions. There is less β-phase in the specimen (approximately three percent of the overall specimen), and its maximum texture, with a texture strength of nine times, is found in Figure 10b and 10d, which correspond to texture types {001} <100> and {110} <$1\bar{1}0$>, respectively. With an 8-fold texture strength, the {001} <110> texture is also visible in Figure 10c and 10f. Due to the low content of β-phase, its texture distribution pattern has less influence on the overall texture distribution of the specimen.

The examination of the texture of A1 in various planes reveals that the specimen is primarily subjected to torsional deformation in the *xy* plane. Following torsional deformation, slip deformation deforms the α-phase, producing the strongest textures, A and B. This type of texture is parallel to the slip system in the column plane, but there are other types of texture with greater strength. At the same time, the β-phase is also twisted, resulting in a weaker deformation texture at {001} <110>.

Since the specimen is in the *xz* and *yz* planes, the axial force increases the β-phase’s susceptibility to transformation in the force’s direction and increases the likelihood that textures will form in the <100> and <110> directions. Conversely, the lower-strength {0001} <2$\bar{1}\bar{1}$0> and {0001} <10$\bar{1}$0> textures form parallel to the {0001} basal slip system because the α-phase is less compressed and more prone to deformation.

**Figure 11:**
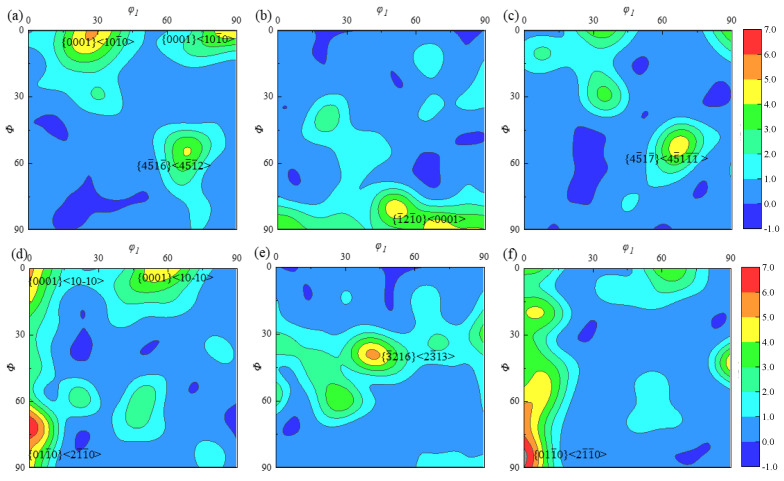
ODF plots of the primary α-phase at typical position A2 in (**a**) *φ*_2_ = 0°, *xz* plane; (**b**) *φ*_2_ = 0°, *yz* plane; (**c**) *φ*_2_ = 0°, *xy* plane; (**d**) *φ*_2_ = 30°, *xz* plane; (**e**) *φ*_2_ = 30°, *yz* plane; and (**f**) *φ*_2_ = 30°, *xy* plane.

Located in the inner part of the specimen, A2 has a weaker overall three-dimensional flow trend than A1; a small rate of temperature decreases after forming, and a uniform distribution of incipient α-phase of larger size and larger number, leading to an average grain size of 11.3 μm. The ODF plots of specimen A2 in three planes, where *φ*_2_ is a constant, are presented in Figure 11 to study the ODF cross sections corresponding to the incipient α-phase at *φ*_2_ = 0° and *φ*_2_ = 30°, and the maximum texture strength is illustrated by red color. As presented in Figure 11a,d, in the *xz* plane, the texture of the incipient α-phase is mainly distributed along {01$\bar{1}$0} <2$\bar{1}\bar{1}$0>, with a maximum texture strength of about 8.15, and there are both {0001} <10$\bar{1}$0> and {4$\bar{5}$1$\bar{6}$} <4$\bar{5}$1$\bar{12}$> textures inside the specimen, with texture strengths of about 5.45 and 4.9. As demonstrated in Figure 11b,e, for the primordial α-phase at the position (40–40–30°), there is a texture of {$\bar{3}$216} <2$\bar{3}$13> in the *yz* plane with a maximum texture strength of 6.77 times. Additionally, {$\bar{1}$2$\bar{1}$0} <0001> texture with a texture strength of roughly 6.14 times exists. The primary α-phase is mostly found in the vicinity of the {01$\bar{1}$0} <2$\bar{1}\bar{1}$0> texture in the *xy* plane, where its maximum strength is approximately 8.16 times. As seen in Figure 11c,f, additional, less intense {4$\bar{5}$1$\bar{7}$} <4$\bar{5}$1$\bar{11}$> texture with a texture intensity of 5.33 times is also dispersed throughout the specimen at the (70°–50°–0°) position.

**Figure 12:**
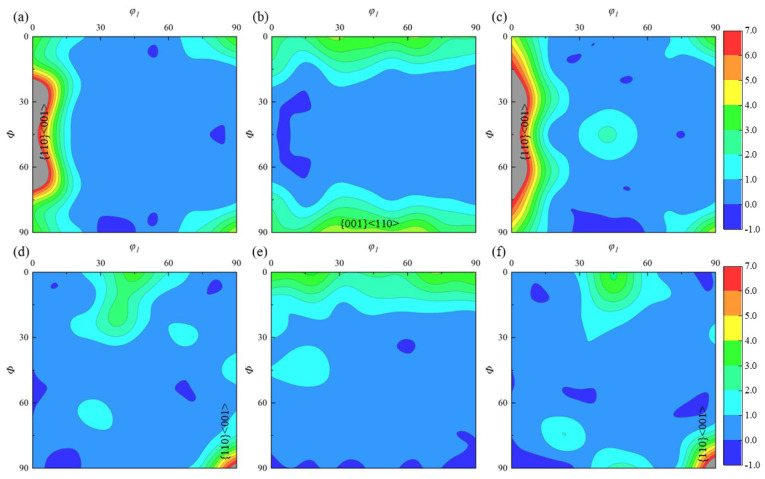
ODF plots of the β-phase at typical position A2 in (**a**) *φ*_2_ = 0°, *xz* plane; (**b**) *φ*_2_ = 0°, *yz* plane; (**c**) *φ*_2_ = 0°, *xy* plane; (**d**) *φ*_2_ = 45°, *xz* plane; (**e**) *φ*_2_ = 45°, *yz* plane; and (**f**) *φ*_2_ = 45°, *xy* plane.

The content of the β-phase at the A2 position after the ACDR deformation is about 1%, and its texture distribution in the three planes is depicted in Figure 12. As for the β-phase structure, at *φ*_2_ = 0°, it is mainly distributed in {110} <001>, with a structure strength of 8.5 and {001} <110> having a structure strength of 4.2, as illustrated in Figure 12a,c. The main {110} <001> texture is present at *φ*_2_ = 45°, with a texture strength of 9.5, as shown in Figure 12d,f, and there is a texture close to {001} <100>, as depicted in Figure 12e, with a maximum strength of 3.8.

**Figure 13:**
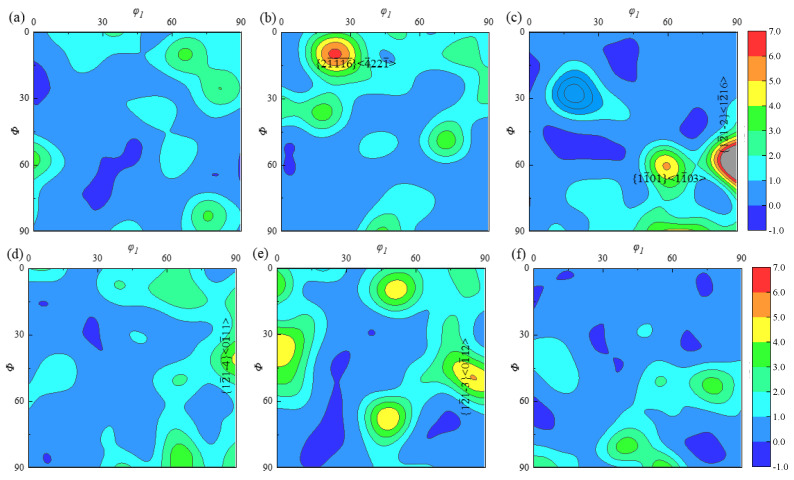
ODF plots of the primary α-phase at typical position A3 in (**a**) *φ*_2_ = 0°, *xz* plane; (**b**) *φ*_2_ = 0°, *yz* plane; (**c**) *φ*_2_ = 0°, *xy* plane; (**d**) *φ*_2_ = 30°, *xz* plane; (**e**) *φ*_2_ = 30°, *yz* plane; and (**f**) *φ*_2_ = 30°, *xy* plane.

As the A3 is located at the edge part of the specimen, the positive strain is small, and the shear strain increases with the distance from the center of the specimen; the incipient α-phase exhibits an elongated ellipsoidal morphology. Due to the temperature drop effect, a particle size slightly smaller than that of A2 is observed, which is about 10.4 μm. Figure 13 displays the ODF plots of the specimen edge position A3 in three planes for the ODF cross sections representative of the incipient α-phase at *φ*_2_ = 0° and *φ*_2_ = 30°, where *φ*_2_ is a constant and red indicates the highest texture intensity. As illustrated in Figure 13a,d, the texture of the incipient α-phase is primarily distributed along (90–40–30°) in the *xz* plane. This corresponds to {1$\bar{2}$1$\bar{4}$} <0$\bar{1}$11> texture, which has a maximum texture strength of 4.9 times. Other texture types are also uniformly distributed within the specimen at the same time. As illustrated in Figure 13b,e, the incipient α-phase is primarily dispersed along the (25–10–0°) {2$\bar{1}\bar{1}\bar{16}$} <$\bar{4}$22$\bar{1}$> texture in the *yz* plane. Within the specimen, there is a texture in the {1$\bar{2}$1$\bar{3}$} <0$\bar{1}$12> direction that has a texture strength of roughly 5.88, and other textures are evenly distributed throughout the specimen, with a strength of 4.5 times. The primary α-phase is primarily present in the *xy*-plane as a texture in the {1$\bar{2}$1$\bar{2}$} <1$\bar{2}$16>, with the strongest at 13.7. This is illustrated in Figure 13c,f. {1$\bar{1}$01} <1$\bar{1}$03> texture with a texture strength of 6.78 times is also present at the (60°–60°–0°) position.

**Figure 14:**
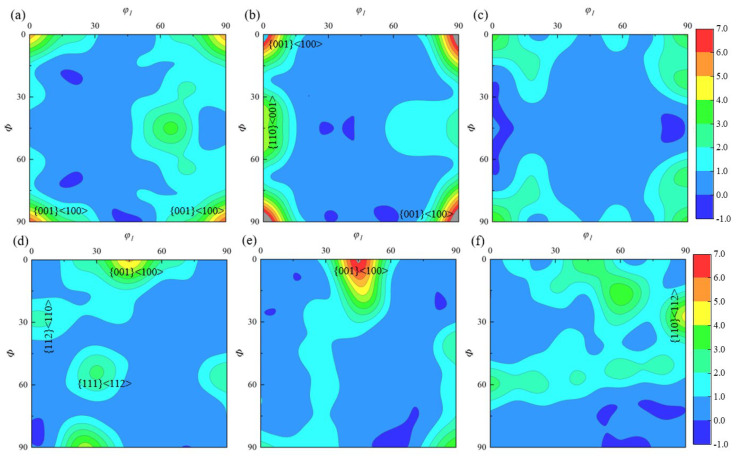
ODF plots of the β-phase at typical position A3 in (**a**) *φ*_2_ = 0°, *xz* plane; (**b**) *φ*_2_ = 0°, *yz* plane; (**c**) *φ*_2_ = 0°, *xy* plane; (**d**) *φ*_2_ = 45°, *xz* plane; (**e**) *φ*_2_ = 45°, *yz* plane; and (**f**) *φ*_2_ = 45°, *xy* plane.

The β-phase content at the A3 position after ACDR deformation is about 2.5%, and its texture distribution in three planes is illustrated in Figure 14. In Figure 14a,b, when *φ*_2_ = 0°, the β-phase texture is mainly distributed in the {001} <100> direction. Its texture density is 9. Moreover, there is a {110} <001> texture with a texture strength of 4.5, as depicted in Figure 14b. In the case of *φ*_2_ = 45°, as depicted in Figure 14e, there is predominantly {001}<100> texture with a strength of 9.5, as well as in the interior of the specimen, where the {110} <112>, {001} <100>, {112} <110>, and {111} <112> textures exist with a texture strength of about 6 times, as illustrated in Figure 14d,f.

In addition to the distribution of textures in the original titanium alloy structure, various types of textures with non-uniform distribution and different strengths were formed after the specimen was subjected to the deformation by ACDR. The compression texture is weaker at the specimen’s edge, and the texture is primarily formed in the *xz* and *yz* planes close to the direction of the column surface. The texture’s strength increases in the *xy* plane, and it gets stronger the closer it is to the specimen’s edge position. At the specimen’s edge, the texture’s strength reaches 13.7, and its direction is mostly dispersed along the cone’s direction.

### 3.3. Room Temperature Tensile Tests and Isotropy Behavior

**Figure 15:**
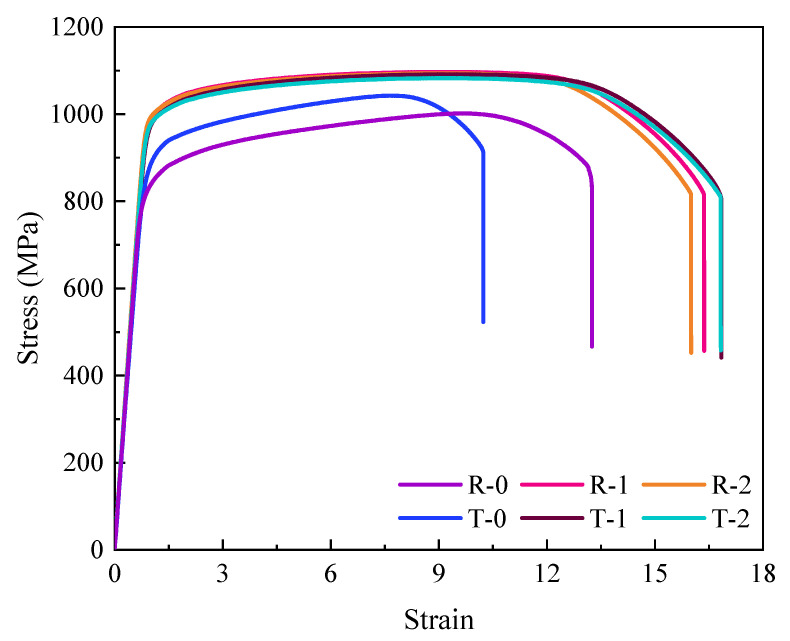
Room temperature tensile curves.

**Figure 16:**
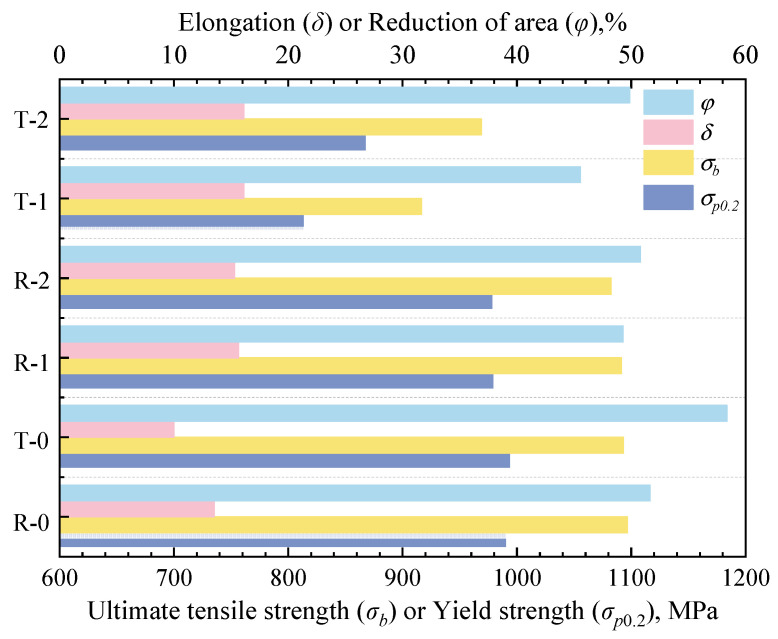
Room temperature tensile data.

Figure 15 displays the TC11 titanium alloy ACDR specimens’ room temperature tensile stress–strain curves, and Figure 16 displays the performance data. The two groups of tensile specimens in different directions have comparatively small differences in yield strength, tensile strength, and elongation, indicating good repeatability. In the ACDR forming process, the average yield strength and tensile strength of the TC11 specimens were 989.01 MPa and 1094.99 MPa, respectively, with a deformation temperature of 980 °C and a deformation amount of 80%. The fine equiaxial organization was formed by spheroidization after the TC11 titanium alloy lamellae’s α-phase fractured during the deformation process. It is possible to increase the specimen strength by decreasing the length of the slip line and decreasing the grain spacing as the size of the primary α-phase decreases.

According to the slip line length, the tensile plasticity of the TC11 titanium alloy is primarily determined by the specimen’s resistance to crack nucleation and extension [21]. The sphericalization fraction of the lamella’s α-phase increases with a decrease in the effective slip line length. This reduces the stress concentration at the α/β-phase interface, makes crack nucleation more challenging, and tends to decrease post-break elongation [22]. Following ACDR molding, the specimen’s primary equiaxial α-phase shrinks in size, resulting in less content and a finer secondary lamellar α-phase. This can better prevent crack extension and create a more zigzagging crack extension path, which raises the TC11 specimen’s fracture shrinkage by roughly 48.9 percent.

**Figure 17:**
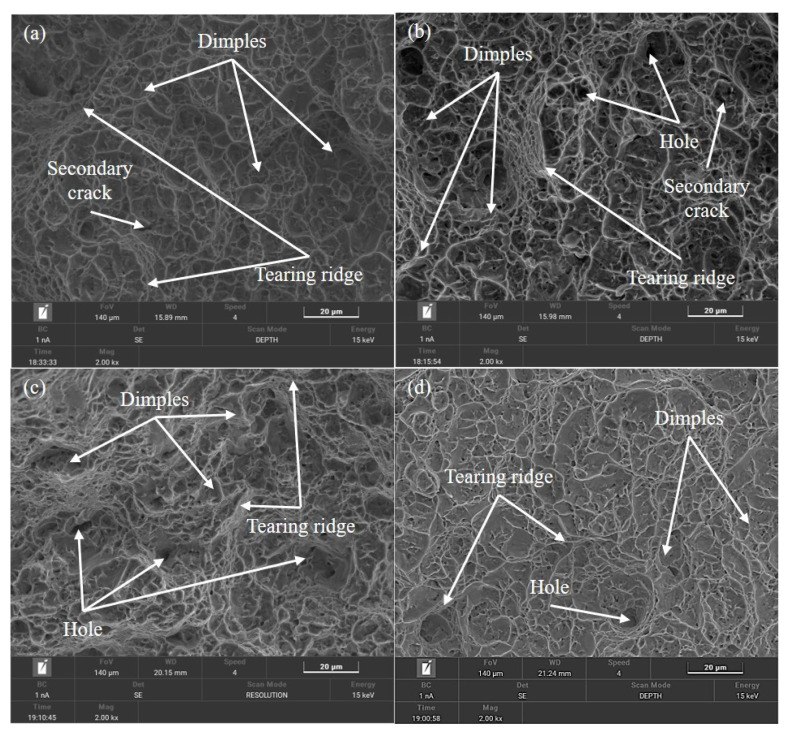
Tensile fracture morphology of the (**a**) R-1, (**b**) R-2, (**c**) T-1, and (**d**) T-2 specimens.

The microstructure of the TC11 titanium alloy specimen’s fiber zone, as evidenced by the tensile fracture in both radial and tangential directions, is illustrated in Figure 17. It is worth noting that the R-1 and R-2 specimens display an increased and larger-sized number of tough fossae in the fracture, typically assuming an equiaxial shape, as presented in Figure 17a,b. The uniform and comprehensive distribution of tear ribs throughout the specimen results in a significant improvement in the tensile strength of the specimen. With a section shrinkage of 50.81 percent, the specimens’ tensile plasticity was considerably enhanced by the presence of secondary cracks of smaller sizes. Tangential specimens’ ligamentous fossae in Figure 17c,d are larger and typically exhibit an elongated state, which is indicative of shear ligamentous fossae. Because of the increasingly evenly distributed tear rib content, the specimen’s tensile strength is likewise high, reaching 1091 and 1082 MPa. Because of the few holes present, the specimen has a slight reduction in tensile plasticity performance and a concentration of local stress. The ligament fossa had an isometric shape in the radial specimen, while the fracture displayed a shear shape in the tangential specimen. The specimen has a much higher tensile strength because there are more and larger tough nests in the fracture, and the tearing ribs are more evenly spaced throughout. As the fracture’s holes decrease and the length and content of secondary cracks gradually decrease; the specimen’s tensile plasticity also increases noticeably.

The tensile characteristics of the disk pieces created by ACDR are noticeably isotropic in both radial and tangential directions, according to the examination of room temperature tensile data and fracture morphology of the TC11 titanium alloy. In both tangential and radial directions, Table 3 provides the isotropy (standard deviation/mean value) of tangential and radial tensile properties in the specimens before and after deformation via ACDR. The specimens of the TC11 titanium alloy exhibit anisotropy in radial and tangential tensile strength of less than 1 percent and tensile plasticity of less than 5 percent, as can be observed. As can be seen from the analysis in Section 3.2, the combined effect of torsion and compression deformation during the ACDR forming process results in specimens with similar texture strengths and different types of texture in both tangential and radial directions. This weakens the orientation relationship of the entire specimen, making the specimens highly isotropic in terms of tangential and radial tensile properties.

## 4. Conclusions

The torsional behavior during ACDR is systematically studied in this paper using numerical simulation tests, and the microstructure orientation distribution of the titanium alloy following ACDR deformation is further studied using EBSD technology. The disk specimens were subjected to room temperature tensile tests following ACDR forming in order to gather information on their tangential and radial performance. Additionally, using SEM technology, the morphology of the titanium alloy specimens’ fractures was described and examined. The following are the key findings.

(1) Since the ACDR is a volume incremental molding technique, strain is progressively accumulated from the specimen’s upper surface downward. Increases in deformation lead to an increase in the specimen’s axial and radial angles as well as the degree of torsion, which enhances the specimen’s strength and plasticity by increasing the amount of strain accumulation in each area.
(2) As can be observed from the examination of the ODF plots, the specimen contains a variety of textures, each with a unique texture strength. The *xz* and *yz* planes are primarily composed of textures {0001} <2$\bar{1}\bar{1}$0> and {01$\bar{1}$0} <2$\bar{1}\bar{1}$0>, which correspond to the strengths of 5.6 and 6.77, respectively, and are formed near the direction of the column plane. The specimen’s edge has a weaker {1$\bar{2}$1$\bar{4}$} <0$\bar{1}$11> texture, with a strength of roughly 4.9. {$\bar{1}$2$\bar{1}$0} <10$\bar{1}$0> and {01$\bar{1}$0} <2$\bar{1}\bar{1}$0> textures with strengths of roughly 8.2 are primarily dispersed along the cone’s direction in the *xy* plane. {1$\bar{2}$1$\bar{2}$} <1$\bar{2}$16> texture has strengths of roughly 13.7, meaning that the closer the specimen’s edge is to the texture’s location, the stronger the texture. Among them, the β-phase is mainly distributed with {001} <100>, {110} <1$\bar{1}$0>, and {110} <001> texture, with approximately 8.5-fold texture strength within the specimen. Nevertheless, because the content of β-phase is too small in the specimen, the distribution pattern of its texture has a weak effect on the texture distribution of the overall specimen.
(3) During the ACDR forming process, compression and torsional deformation occur simultaneously, resulting in the formation of uneven texture strengths and weakening the overall orientation of the specimen relationship. As a result, the specimen’s tensile properties exhibit high levels of anisotropy in all directions, with tensile strength and plasticity anisotropy exceeding 99 percent and 95 percent, respectively.

## Figures and Tables

**Table 1:** The main components of the TC11 alloy.

Elements	Ti	Al	Mo	Zr	Si	Fe	C
Content (wt%)	Bal	6.92	3.3	1.6	0.35	0.03	0.05
Standard (wt%)	Bal	5.8~7	2.8~3.8	0.8~2	0.2~0.35	≤0.25	≤0.1

**Table 2:** ACDR deformation test program.

Parameters	ACDR
Billet size (Diameter (Φ) × Height (d) (mm))	Φ60 × 60
Positioning table size (Φ × d (mm))	Φ40 × 10
Heat temperature of the billet (°C)	980 (Tβ − 30)
Preheating temperature of the dies (°C)	200
Axial reduction (%)	80
Rotation speed of the lower die (r/min)	35
Inclination angle of the upper die (°)	6
Feed speed of the upper die (mm/s)	2.5
Mold materials	42CrMo

**Table 3:** Isotropy of the tensile properties in different directions.

Processes	Yield Strength/MPa	Ultimate Tensile Strength/MPa	Uniform Elongation/%	Reduction of Area/%
Original	3.23%	2.77%	14.94%	5.80%
ACDR	0.69%	0.43%	2.15%	4.10%

## Data Availability

The original contributions presented in this study are included in the article. Further inquiries can be directed to the corresponding author.
